# Hip Structural Parameters over 96 Weeks in HIV-Infected Adults Switching Treatment to Tenofovir-Emtricitabine or Abacavir-Lamivudine

**DOI:** 10.1371/journal.pone.0094858

**Published:** 2014-04-10

**Authors:** Hila Haskelberg, Nicholas Pocock, Janaki Amin, Peter Robert Ebeling, Sean Emery, Andrew Carr, STEAL study investigators, Anthony Allworth, Jonathan Anderson, David Baker, Mark Bloch, Mark Boyd, John Chuah, David Cooper, Stephen Davies, Linda Dayan, William Donohue, Nicholas Doong, Dominic Dwyer, John Dyer, Robert Finlayson, Michelle Giles, David Gordon, Mark Kelly, Nicholas Medland, Richard Moore, David Nolan, David Orth, Jeffrey Post, John Quin, Tim Read, Norman Roth, Darren Russell, David Shaw, David Smith, Don Smith, Alan Street, Ban Kiem Tee, Ian Woolley

**Affiliations:** 1 The Kirby Institute, University of New South Wales, Sydney, Australia; 2 St. Vincent's Hospital, Sydney, Australia; 3 North West Academic Centre, University of Melbourne, Melbourne, Australia; Harvard Medical School, United States of America

## Abstract

**Background:**

Therapy with tenofovir is associated with lower bone mineral density (BMD), higher markers of bone turnover and increased fracture risk in HIV-infected adults. Bone structural parameters generated by hip structural analysis may represent a separate measure of bone strength, but have not been assessed in HIV.

**Methods:**

Dual-energy X-ray absorptiometry (DXA) scans from 254 HIV-infected adults randomised to simplify their existing dual nucleoside analogue reverse transcriptase inhibitor therapy to coformulated tenofovir-emtricitabine or abacavir-lamivudine were analysed using DXA-derived hip structural analysis software. Hip structural parameters included femoral strength index, section modulus, cross-sectional area, and cross-sectional moment of inertia. We used one-way ANOVA to test the relationship between nucleoside analogue type at baseline and structural parameters, multivariable analysis to assess baseline covariates associated with femoral strength index, and t-tests to compare mean change in structural parameters over 96 weeks between randomised groups.

**Results:**

Participants taking tenofovir at baseline had lower section modulus (−107.3 mm^2^, p = 0.001), lower cross-sectional area (−15.01 mm^3^, p = 0.001), and lower cross-sectional moment of inertia (−2,036.8 mm^4^, p = 0.007) than those receiving other nucleoside analogues. After adjustment for baseline risk factors, the association remained significant for section modulus (p = 0.008) and cross-sectional area (p = 0.002). Baseline covariates significantly associated with higher femoral strength index were higher spine T-score (p = 0.001), lower body fat mass (p<0.001), lower bone alkaline phosphatase (p = 0.025), and higher osteoprotegerin (p = 0.024). Hip structural parameters did not change significantly over 96 weeks and none was significantly affected by treatment simplification to tenofovir-emtricitabine or abacavir-lamivudine.

**Conclusion:**

In this population, tenofovir use was associated with reduced composite indices of bone strength as measured by hip structural analysis, but none of the structural parameters improved significantly over 96 weeks with tenofovir cessation.

**Trial Registration:**

ClinicalTrials.gov NCT00192634

## Introduction

Low bone mineral density (BMD) and higher rates of fractures have been reported in HIV-infected adults compared with general population controls [1], [2], and have been particularly associated with tenofovir (TDF) therapy [3]. BMD as measured by dual-energy X-ray absorptiometry (DXA) is the gold standard for clinical assessment of bone fragility. The relationship between decreased BMD and increased risk of fractures is well established [4]. BMD, however, only accounts for about 50% of fracture risk [5] and does not describe other components of bone quality such as geometric configuration, which can be estimated by DXA-derived hip structural analysis (HSA) [6], [7]. HSA software generates a femoral strength index, an integral measure that combines BMD, femur geometry, age, height and weight and aims to reflect the bone's ability to withstand forces generated during a fall [8].

A cross-sectional analysis of hip osteoporotic fractures and composite indices of femoral neck strength in healthy women found decreased measures of femoral neck strength in women with fractures [9]. In addition, structural parameters were found to predict hip fracture in postmenopausal women after adjusting for both clinical risk factors and BMD [10]–[13]. Yet the role of these measures as independent predictors of hip fracture remains controversial, particularly in men [14].

Studies of osteoporosis and fractures in HIV-infected adults have mainly focused on BMD; the effects of HIV and its treatment on other measures of bone quality are unclear. Walker Harris et al. [15] have recently found in a cross-sectional study that HIV/HCV-co-infected men had significantly lower measures of hip strength at the narrow neck and shaft when compared to healthy controls. Lower lean body mass accounted for most of the differences between groups after adjusting for race, age, smoking status, height, and weight [15]. In a longitudinal study of perinatally HIV-infected youth (n = 31; 9–18 y), neither bone geometry nor strength was significantly different compared with healthy controls [16].

In the STEAL study, patients randomized to simplify their existing dual nucleoside reverse transcriptase inhibitor (NRTI) therapy to coformulated tenofovir-emtricitabine (TDF-FTC) had greater BMD decreases and greater bone turnover marker increases over 96 weeks than those who were randomised to abacavir-lamivudine (ABC-3TC) [17]. STEAL provides an opportunity to examine bone structure cross-sectionally and longitudinally, and in particular any effect of TDF. We hypothesised that there would be a significant difference between TDF-FTC and ABC-3TC in measures of bone structure, as assessed by DXA-derived Hip Structural Analysis. The aim of this analysis was to estimate and compare changes in bone structural parameters by randomised arm from baseline to 96 weeks. A secondary objectives was were to determine the relationship between femoral strength index and baseline clinical and biochemical characteristics, and to explore the relationship between markers of bone turnover and changes in the above measures of bone structure

## Methods

### Study design

STEAL was an open-label, prospective, randomized, non-inferiority study that compared simplification of current NRTIs to fixed-dose combination TDF-FTC or ABC-3TC over 96 weeks in 357 adults with plasma HIV viral load <50 copies/mL [17]. The supporting CONSORT checklist and STEAL protocol are available as supporting information; see Checklist S1, and Protocol S1

### Ethics Statement

The study was approved by each site's Human Research and Ethics Committee and registered at Clinicaltrials.gov (NCT00192634). The specific ethics committees that gave approval for the STEAL study are: St Vincent's Hospital Human Research Ethics Committee (HREC), South Eastern Sydney/Illawarra Area Health Service HREC, Harbour HREC of Northern Sydney Central Coast Health, North Coast Area Health Service HREC, Sydney West Area Health Service HREC, Sydney South West Area Health Service HREC, Alfred Hospital EC, Southern Health HREC, Melbourne Health HREC, The Prince Charles Hospital HREC, Cairns & Hinterland Health Service District EC, Gold Coast Health Service District HREC, Royal Brisbane and Women's Hospital HREC, Flinders Clinical Research EC, Royal Adelaide Hospital Research EC, Royal Perth Hospital EC. Each participant signed a written informed consent before enrolment.

### DXA and hip structural analysis

DXA measurements of the right hip and lumbar spine BMD were performed for each participant at baseline, week 48, and week 96, using a standardized protocol. The following were recorded: hip and spine BMD, T-scores and Z-scores, total body fat tissue and lean tissue masses. DXA instruments varied between study sites and for this analysis, only data from GE-Lunar scanners were used.

Archived lunar image files of the right femur were analysed by a trained individual blinded to antiretroviral therapy, using the HSA software Lunar Prodigy enCORE 2011 version 13.60.033 (GE Healthcare). The HSA software provides a line of pixels traversing the bone axis which gives a projection of the surface area of bone in the cross section. The results reported in this analysis are from the femoral neck. The HSA software automatically assesses all cross sections in the femoral neck and identifies the plane that is the weakest, by calculating the mass distribution derived from an X-ray absorption curve [8]. Specifically, the following structural parameters were obtained: hip axis length (HAL, mm), femoral strength index, buckling ratio, section modulus (Z, in mm^3^), cross-sectional moment of inertia (CSMI, in mm^4^), and cross sectional area (CSA, in mm^2^) (Table 1).

**Table 1:** Hip structural parameters included in this analysis [8].

Measure	Derivation	Comments
Femoral strength index	Calculated as strength/stress, where, strength = 185–0.34(age - 45) and stress is moment*y/CSMI+force/CSA	Indicates the bone's resistance to fracture from forces generated during a fall on the greater trochanter
Hip axis length (HAL, mm)	The distance along the femoral neck axis, extending from the bone edge at the base of the trochanter to the bone edge at the inner pelvic brim	Suggested to be a measure of the degree to which the femur extends beyond the pelvis, increasing risk for impact, though relationship to fractures is unclear
Buckling ratio	Ratio of the outer radius to the cortical thickness	Indicator of cortical stability under compressive loads
Section Modulus (Z, mm^3^)	CSMI divided by half the width of the femoral neck	A measure of bending strength
Cross-sectional area (CSA; mm^2^)	Represents the area of mineral packed together in the defined cross section of the femoral neck	Proportional to bone's ability to resist axial compressive force
Cross-sectional moment of inertia (CSMI; mm^4^)	calculated using the mass distribution derived from the absorption curve	Bone's ability to resist bending forces

### Laboratory markers

Plasma and serum samples were collected at baseline and at weeks 12, 24, 48, 72 and 96 (following a 10-hour overnight fast, except at week 12) and stored at −70°C. Markers of bone resorption (C-terminal cross-linking telopeptide of type 1 collagen [βCTX]), bone formation (procollagen type 1 N-terminal propeptide [P1NP]; bone-specific alkaline phosphatise [BALP]) and regulators of bone turnover (osteoprotegerin [OPG] and receptor activator of nuclear factor kappa ligand [RANKL]) were evaluated. The following were assessed at baseline only: interleukin-6, oestradiol, free testosterone and 25-hydroxy vitamin D. Assays are described elsewhere [18]. BTMs were batch-tested after study completion in one laboratory.

### Statistical analyses

All analyses were performed on the per-protocol data so as to evaluate the biological effects of exposure to randomised therapy. “Per-protocol” is defined as available data collected on participants while on randomised strategy as defined in the STEAL protocol.

The associations between baseline covariates (including demographic, HIV-related factors, antiretroviral therapy, body composition, bone remodelling regulators, sex hormones, and vitamin D), and absolute baseline femoral strength index were analysed using linear regression. Multivariable model was built using backward, stepwise methods. Covariates that achieved a p-value <0.1 in univariate analysis were assessed for inclusion in the model. In an exploratory analysis, one-way ANOVA was used to assess the relationship between type of NRTI at study entry (ABC, TDF, or other) and all structural measures at baseline. To test this relationship further, linear regression models were a-priori adjusted for covariates known to be related to bone status i.e. age, sex, ethnicity, smoking, height, total body fat mass, total body lean mass, and HIV infection duration to account for possible associations with hip structural analysis outcomes. Structural parameters at week 48 and week 96 were compared with baseline using paired t-tests. Randomized groups were compared for changes in structural measures by t-tests at 48 weeks and 96 weeks. Results are reported as regression coefficients i.e. differences between groups. Statistical significance was defined as a 2-sided α of 0.05. Statistical analyses were performed with STATA (StataCorp. 2011. Stata Statistical Software: Release 12. College Station, TX: StataCorp LP USA).

## Results

**Figure 1:**
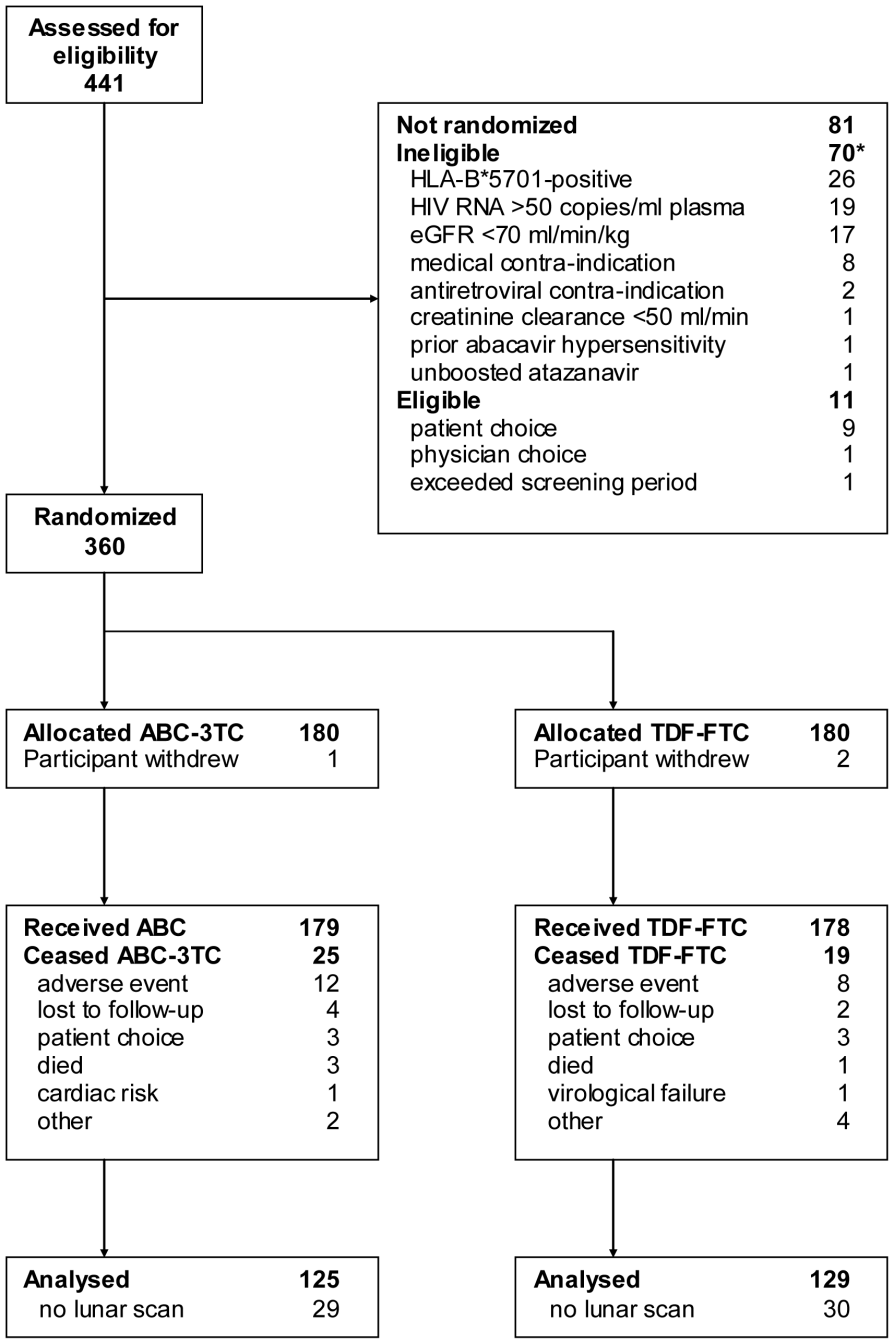
Patient disposition.

Patient disposition is outlined in Figure 1. Of 357 participants enrolled in the parent study, 285 had GE-lunar Prodigy scans data available at baseline; 17 discontinued ABC-3TC and 14 discontinued TDF-FTC by week 96. Therefore, the analysed per-protocol population comprised the remaining 254 participants (71% of main study population). Baseline characteristics of the population analysed were similar to those of all study participants [19] and well balanced between arms (Table 2). At study entry, 29% of the participants (n = 74) were taking TDF-containing regimen, 21% (n = 53) were on ABC-containing regimen and 50% (n = 127) were taking other NRTIs (typically two of the following: 3TC, FTC, zidovudine, didanosine, stavudine).

**Table 2:** Baseline characteristics.

	NRTI at study entry			Randomization		
Baseline characteristics	ABC (n = 53)	TDF (n = 74)	Other (n = 127)	ABC-3TC (n = 125)	TDF-FTC (n = 129)	Main Study (n = 357)
Age (years)	48.7±8.4	42.8±8.5	44±8.4	45.8±8.9	44.3±8.4	45.1±8.6
Male (%)	98	99	98	99	98	98
Ethnicity – white (%)	92	88	79	84	85	86
HIV duration (years)	13.4±5.6	8.7±5.7	9.9±5.7	10.0±5.7	10.5±6.1	10.2±6.0
CD4+ count	613±257	609±257	612±248	629±259	595±245	612±282
**ART exposure**						
NRTI duration (years)	8.0±4.1	4.1±3.1	5.8±2.8	5.8±3.5	5.8±3.5	5.8±3.6
Current protease inhibitor (%)	26	28	19	24	22	23
**Anthropometric factors**						
Weight (kg)	76.8±11.5	72.9±12.2	77.7±12.8	76.0±12.9	76.2±12.2	76.8±12.6
Fat mass (kg)	16.2±6.8	14.9±7.2	16.5±7.7	15.7±7.3	16.2±7.5	16.8±7.9
**Bone mineral density**						
Right hip (g/cm^2^)	1.06±0.14	1.00±0.11	1.04±0.13	1.04+0.13	1.02+0.13	
Lumbar Spine (g/cm^2^)	1.26±0.14	1.16±0.15	1.21±0.16	1.2±0.16	1.2±0.15	
**Bone turnover markers**						
βCTx (ng/L)	256±152	314±157	226±134	252±154	265±145	
BALP (µg/L)	16.8±8.4	23.6±11.6	17.9±10.3	19.3±10.1	19.4±11.2	
P1NP (µg/L)	45.7±19.1	67.7±22.8	50.1±21.9	53.1±23.9	55.7±22.6	
OPG (pmol/L)	3.8±1.1	4.0±1.4	3.8±1.1	3.9±1.3	3.8±1.1	
RANKL (pmol/L)	0.2±0.3	0.2±0.2	0.3±0.5	0.2±0.3	0.2±0.4	
**Hip structural parameters**						
Femoral strength index	1.68±0.43	1.57±0.34	1.60±0.38	1.60±0.37	1.62±0.39	
Hip axis length (mm)	118.1±6.9	116.8±7.1	118.9±8.5	118.2±7.4	118.1±8.2	
Buckling ratio	3.9±1.6	4.4±1.5	4.2±1.8	4.2±1.7	4.2±1.7	
Section modulus (mm^3^)	873±196	766±147	836±180	832±175	815±182	
Cross-sectional area (mm^2^)	174±28	159±20	169±26	167±25	167±26	
Cross-sectional moment of inertia (mm^4^)	16235±5032	14198±3387	15705±4222	15473±4127	15283±4368	

**Note.** Results are expressed as mean ± standard deviation or %.

Abbreviations: **ABC-3TC**, abacavir-lamivudine; **BALP**, bone-specific alkaline phosphatase; **βCTx**, C-terminal cross-linking telopeptide of type 1 collagen; **BMD**, bone mineral density; **NNRTI**, non-nucleoside reverse transcriptase inhibitor; **NRTI**, nucleoside reverse transcriptase inhibitor; **OPG**, osteoprotegerin; **P1NP**, procollagen type 1 N-terminal propeptide; **RANKL**, Receptor Activator of Nuclear Factor Kappa Ligand; **TDF-FTC**, tenofovir-emtricitabine.

### Hip structural parameters at baseline

Baseline CSMI, CSA, and section modulus were all significantly different across the three sub-groups of NRTI types at baseline [CSMI: *F*(2,208) = 26.98, p = 0.013; CSA: *F*(2,251) = 6.59, p = 0.002; section modulus: *F*(2,251) = 6.51, p = 0.002].

**Figure 2:**
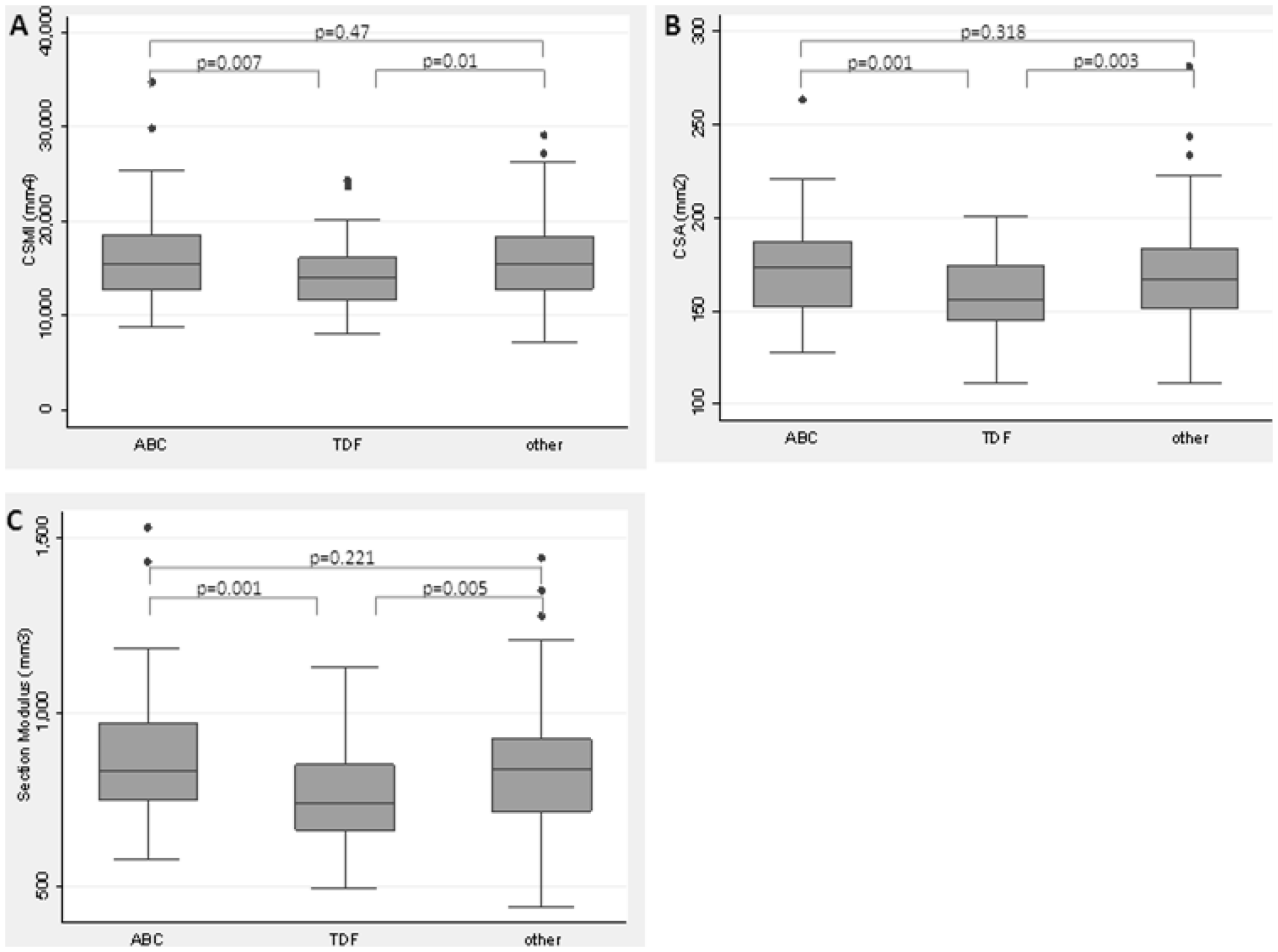
Hip structural parameters at baseline by type of NRTI at study entry (A) CSMI (bone's ability to resist bending), (B) CSA (bone's ability to resist axial compressive force) and (C) section modulus (bending strength). Abbreviations: CSA, cross-sectional area; CSMI, cross-sectional moment of inertia.

Compared to the ABC group, participants that entered the study on TDF had lower baseline CSMI (coefficient: −2036.8 mm^4^; 95% CI: −3520.8 to −552.7; p = 0.007), lower CSA (−15.01 mm^2^; 95% CI: −23.8 to −6.2; p = 0.001), and lower section modulus (−107.3 mm^3^; 95% CI: −169.1 to −45.4; p = 0.001) (Figure 2). After adjustment, the association remained significant for CSA (−13.7 mm^2^; 95% CI: −21.3 to −6.2; p = 0.002) and section modulus (−83.1 mm^3^; 95% CI: −135.5 to −30.6; p = 0.008), but not for CSMI (−1168.85 mm^4^; 95% CI: −2366.9 to 29.2; p = 0.159).

Baseline covariates significantly associated with higher femoral strength index at baseline in multivariable analysis were higher spine T-score (p trend = 0.001), lower body fat mass (p trend<0.001), lower bone alkaline phosphatase (BALP; p = 0.025), and higher osteoprotegerin (OPG; P = 0.024; Table 3).

**Table 3:** Covariates associated with femoral strength index at baseline

Covariate	Univariate analysis					Multivariate model (n = 228)			
	N	Coef. femoral strength index	95% Conf. Interval	P>|z|	overall P value	Coef. femoral strength index	95% Conf. Interval	P>|z|	overall P value
HIV RNA (copies/ml)	254	0	(−0.00 to 0.00)	0.09		0	(−0.00 to 0.00)	0.191	
No PI - NNRTI*	195	0				0			
PI	59	−0.12	(−0.23 to −0.01)	0.027		−0.09	(−0.20 to 0.01)	0.079	
Spine BMD wk0 - quartiles									
0.788–1.061*	54	0				0			
1.062–1.180	60	0.17	(0.03 to 0.31)	0.016		0.15	(0.01 to 0.28)	0.034	
1.181–1.292	71	0.22	(0.09 to 0.35)	0.001		0.27	(0.14 to 0.40)	<0.001	
1.293–1.798	69	0.2	(0.06 to 0.33)	0.004	0.005	0.18	(0.06 to 0.31)	0.005	**0.003**
Total body lean tissue mass wk0 - quartiles									
32464–51980 g*	67	0				0			
51981–56673 g	57	0	(−0.13 to 0.14)	0.977		0.03	(−0.10 to 0.16)	0.677	
56674–61811 g	62	−0.1	(−0.23 to 0.03)	0.129		−0.05	(−0.18 to 0.08)	0.436	
61812–79671 g	68	−0.09	(−0.21 to 0.04)	0.191	0.09	−0.04	(−0.17 to 0.10)	0.614	0.391
Total body fat tissue mass wk0 - quartiles									
1110–10590 g*	69	0				0			
10591–15428 g	59	−0.17	(−0.29 to −0.04)	0.008		−0.19	(−0.32 to −0.07)	0.002	
15429–20942 g	64	−0.25	(−0.37 to −0.13)	<0.001		−0.31	(−0.43 to −0.19)	<0.001	
20943–46433 g	62	−0.36	(−0.48 to −0.24)	<0.001	<0.001	−0.38	(−0.50 to −0.25)	<0.001	**<0.001**
Alkaline phosphatase (mmol/l)	253	0	(−0.00 to −0.00)	0.039		0	(−0.00 to 0.00)	0.886	
BALP Wk0	228	0	(−0.01 to 0.00)	0.066		−0.01	(−0.01 to −0.00)	**0.03**	
OPG Wk0	228	0.04	(0.00 to 0.09)	0.032		0.04	(0.00 to 0.08)	**0.038**	
RANK-L Wk0	228	0.15	(0.02 to 0.28)	0.021		0.13	(0.01 to 0.25)	**0.032**	

### Changes in hip structural parameters over 96 weeks

For all structural measures besides HAL, there was no significant difference between baseline and week 48 or between baseline and week 96 for the whole cohort. The absolute increase in HAL from baseline was statistically significant at week 48 though not at week 96 [(diff: 0.25; 95% confidence intervals [CI]: 0.09 to 0.41; p = 0.002); (diff: 0.16; 95% CI: −0.005 to 0.33; p = 0.058, respectively]. There was no difference between randomised arms in absolute change at week 48 or week 96 (Table 4).

**Table 4:** Mean change in hip structural parameters over 96 weeks by randomisation

Hip structural parameters	Abacavir-lamivudine	Tenofovir-emtricitabine	Mean difference (95% confidence interval)	*P*
Femoral strength index	−0.02	−0.01	0.01 (−0.05 to 0.07)	0.704
Hip axis length (mm)	0.3	0.03	−0.27 (−0.6 to 0.6)	0.109
Buckling ratio	−0.06	−0.3	−0.2 (−0.7 to 0.1)	0.220
Section modulus (mm^3^)	2.2	0.2	−2.0 (−16.8 to 12.7)	0.787
Cross-sectional area (mm^2^)	1.3	−0.2	−1.5 (−3.8 to 0.6)	0.155
Cross-sectional moment of inertia (mm^4^)	−42.4	−3.8	38.6 (−276 to 353)	0.810

Analysing the effect of randomisation by the NRTI sub-groups, patients that were on ABC at baseline and switched to TDF (n = 24) had significantly greater decreases of CSA (coeff: −6.7 mm^2^; 95% CI: −12.3 to −1.0; p = 0.021) and CSMI (coeff: −675.1 mm^4^; 95% CI: −1298.4 to −51.7; p = 0.034) at week 48 compared with those who continued on ABC (n = 26). There were no significant differences from baseline to week 96. No significant change was found for 74 patients on TDF at baseline who either stayed on TDF or switched to ABC.

## Discussion

To our knowledge, this is the first longitudinal study to investigate DXA-derived structural properties of the femoral neck in HIV-infected adults. In this cohort of predominantly young HIV-infected men, participants that were taking TDF at study entry had decreased composite indices of bone strength, as estimated by hip structural analysis, compared to ABC and other NRTIs. Independent baseline factors associated with lower femoral hip strength index at baseline included lower bone formation markers and lower body fat mass. The majority of structural parameters did not improve over 96 weeks of follow-up and nor were they significantly affected by treatment simplification to TDF-FTC or ABC-3TC.

Most studies of HIV-infected adults have shown that they are at greater risk of fractures when compared with healthy population controls [1], [2], yet the pathogenesis of HIV-associated bone disease is multifactorial and not fully understood. In the STEAL cohort, four participants experienced fractures, two in each treatment arm [17]. In a number of randomized controlled studies, the initiation or a switch to ART was associated with 6–12 months of bone loss, which then stabilizes [17], [19]. In post-menopausal women, treatment with a bisphosphonate resulted in significant improvements in hip geometric parameters after one [20] and two years [21] of treatment. The finding in our cohort that most of the structural indices did not significantly change following ART switch, supports the finding by Tuck et al. that some bone geometrical measures are relatively stable over time in men [14]. Furthermore, our data also corroborate a recent population-based study (n = 1760), which found that a decline in BMD was counteracted by an increase in bone size, resulting in only a small decrease of up to 0.5% in composite indices of hip strength (as measured by DXA-derived hip structural analysis), resulting in a partial preservation of bone strength in men from peak value to age 90 years [22].

In our study, higher OPG was independently associated with higher femoral strength index. The RANKL/RANK/OPG signalling pathway has a critical role in bone remodelling; specifically, OPG inhibits bone resorption [23]. Our results are consistent with the Framingham Offspring Study in HIV-uninfected adults (n = 1165 men), which found that increased OPG was independently positively associated with indices of hip strength in men [24]. Higher OPG levels may reflect a compensatory reaction to accelerated bone resorption and deterioration of cortical bone [25]. This may also explain the observation that there were no significant changes over the 2 years of follow-up in majority of structural parameters in our cohort. We also found that lower body fat mass was an independent predictor of higher femoral strength index, which is consistent with the index's formula – negative correlation between the strength (resistance to fracture forces) relative to load (forces placed on the hip during a fall).

Unlike the previous cross-sectional study of hip geomerty in HIV/HCV co-infected adults [15], we found no association between any of the structural parameters and lower lean mass. However, it is difficult to directly compare the two studies. Firstly, chronic hepatitis C monoinfection has been independently associated with unbalanced bone turnover and reduced bone quality [26]. Secondly, the population studied by Walker-Harris was mostly African-American (86%) while 85% of our cohort was Caucasian and probably less than 10% had HCV co-infection. And finally, the participants in the HIV/HCV cohort were evaluated at three locations at the proximal femur using a Hologic scanner, which employs a different HSA method to assess strength at the femoral neck than the Lunar software used in our study.

Despite being younger with shorter duration of HIV and shorter exposure to ART, participants that were on TDF at study entry had significantly lower CSA (bone's ability to resist bending) and section modulus (bending strength) at baseline than those receiving other NRTIs. Switching from TDF lead to an increase in BMD [27], in our study, however, TDF cessation did not lead to a significant improvement in any structural parameter of the hip over 96 weeks. The Study of Osteoporotic Fractures (n = 7474) found that that a 1 SD decrease in CSA increased the risk of incident hip fracture by 1.80–1.93, depending on which covariates were included in the model [13]. There is a body of literature demonstrating a greater effect of TDF-based regimens on decreasing BMD [17], [19], [28] and increased fracture risk [3]. As the reduced composite indices of bone strength that were found with TDF, did not improve after treatment switch, it may imply that prevention of loss in bone strength is more important than switching treatment to improve these bone measures. It was recently found by Bedimo at el. [3] that cumulative exposure to TDF was an independent predictor of increased risk of osteoporotic fractures (yearly HR 1.12; 95% CI 1.03–1.21). The possible effects of long-term exposure to TDF on bone structure require further investigation in patients initiating antiretroviral therapy.

Our study has several limitations. There is an inherent limitation in using DXA, which produces two-dimensional images, to assess three-dimensional measures of hip geometry and the calculated strength indices [6]. Furthermore, relative changes in the cortical versus cancellous bone are not detected by DXA. However, the DXA-derived HSA method is currently more available and affordable than the three-dimensional techniques (such as finite element modelling), and allows the evaluation of femoral neck structure in additional to BMD. Our finding regarding the association to TDF was only found in the uncontrolled cross-sectional analysis at baseline. Another limitation is the generalizability of our findings: our cohort comprised mainly Caucasian men and we only analysed data from GE-Lunar scanners, therefore limiting our ability to extend our findings to different populations or to other methods that assess hip strength. Lastly, the short follow-up period and small sample size precluded the investigation of fracture risk. Nevertheless, our study includes a comprehensive set of bone-related data - bone turnover markers, BMD and hip structure, which allows us a greater understanding of the skeletal status of this cohort.

To conclude, HIV-infected adults who were on TDF at study entry, had reduced composite indices of bone strength (as assessed by hip structural analysis), compared with other NRTIs groups. Treatment simplification to TDF-FTC or ABC-3TC had no significant effect on hip structural parameters over 96 weeks of follow-up. This study suggests that hip structural parameters may not improve with TDF cessation in our cohort's age group and their assessment does not add any predictive value to BMD in clinical management of HIV-infected adults. Yet, differences in the length, width, and angle of the femoral neck may increase fracture risk with ageing; further longer prospective studies of bone structure in larger cohorts of individuals with HIV may shed more light on the pathogenesis of bone disease in HIV-infection.

## Supporting Information

Checklist S1 **The supporting CONSORT checklist.**
(PDF)

Protocol S1 **STEAL main study protocol.**
(PDF)
